# Pembrolizumab-Related Side Effects: Acute Renal Failure and Severe Neurological Toxicity

**DOI:** 10.3390/medicina58020209

**Published:** 2022-01-30

**Authors:** Gabriele Fasano, Ingrid Marcela Pabon, Yaroslava Longhitano, Christian Zanza, Graziano Carlidi, Enrico Ravera, Andrea Della Selva

**Affiliations:** 1 Department of Emergency Medicine, Anesthesia and Critical Care Medicine, Michele and Pietro Ferrero Hospital, 12060 Verduno, Italy; gabriele.fasano.89@gmail.com (G.F.); paboningrid@hotmail.com (I.M.P.); lon.yaro@gmail.com (Y.L.); gcarlidi@libero.it (G.C.); eravera@aslcn2.it (E.R.); andreadellaselva@libero.it (A.D.S.); 2 Research Training Innovation Infrastructure, Research and Innovation Department, Azienda Ospedaliera SS Antonio e Biagio e Cesare Arrigo, 15121 Alessandria, Italy; 3 Foundation of “Ospedale Alba-Bra”, 12060 Verduno, Italy

**Keywords:** pembrolizumab, adverse reaction, pembrolizumab toxicity, renal toxicity, neurological toxicity

## Abstract

Immunotherapy with immune checkpoint inhibitors represents nowadays a marked improvement in cancer treatment. Nevertheless, they can cause severe toxicities that put the patient at high risk, often requiring aggressive treatment. We present the case of a female patient who developed a severe immune-related adverse reaction to Pembrolizumab prescribed for melanoma treatment. Her array of symptoms, which presented a few days after last drug administration, consisted of severe neurological deficit, severe renal failure, polymyositis, and hyperthyroidism. Treatment required the immediate interruption of the trigger drug, infusion of high dose steroids, renal replacement therapy, plasmapheresis, and methimazole, as will be further discussed.

## 1. Background

Immunotherapy with immune checkpoint inhibitors nowadays represents a marked improvement in cancer treatment: for example, antibodies anti-CTLA4, anti-PD1, and anti-PDL1 have been approved for advanced melanoma and other cancers [1]. We will focus on the mechanism of action of Pembrolizumab, the key element of our case report.

It is not unusual that tumors develop the ability to evade the immune response, increasing the expression of a transmembrane protein called Programmed Cell Death Protein Ligand 1 (PDL1) [2,3]. This protein links to another protein called Programmed Cell Death Protein 1 (PD1) expressed on activated T cells (and on B cells, dendritic cells, and NK cells) and begins an intracellular cascade signaling that results in the inhibition of antitumoral T cells [3].

**Figure 1:**
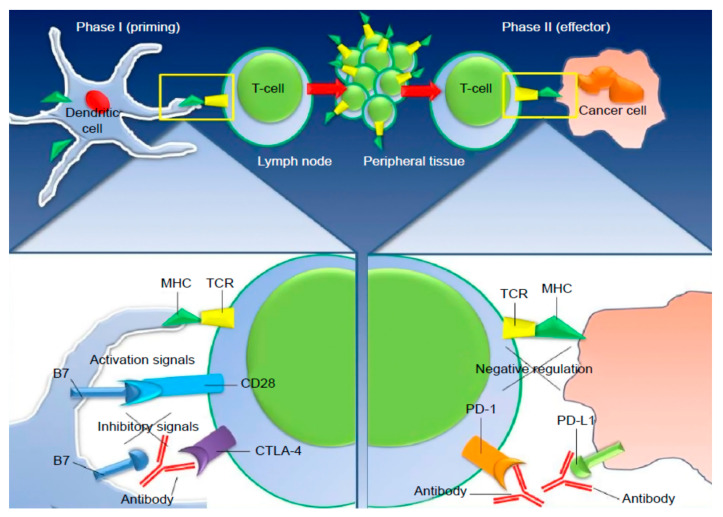
CTLA-4 and PD-1 inhibitor mechanism after the activation of T-cells through their “primed” T-cell receptor, as well as a co-regulatory signal delivered by the B7 family of receptors (immune checkpoints). Notes: CTLA-4, up-regulated shortly after activation, negatively regulates T-cell activation, binding to B7 molecules on APCs surface, during the priming phase of T-cell response within the lymph nodes. B7 molecules binding to CD28, instead, generates the opposite, activating signals. During the effector phase of T-cell immune-response, PD-1 is expressed on T-cells and binds to either of its ligands (PD-L1 or PD-L2), which are primarily expressed within inflamed tissues and the tumor microenvironment, resulting in inhibition of T-cell activity. Antibodies direct to CTLA-4 or PD-1/PD-L1 can activate T-cells with specificity for cancer cells. Abbreviations: MHC, major histocompatibility complex; PD-1, programmed cell death-1; CTLA-4, cytotoxic T-lymphocyte antigen-4; TCR, T-cell receptor.

Pembrolizumab is a monoclonal antibody IgG4 kappa anti-PD1 that promotes the immune response against cancer through this very mechanism (Figure 1) [1]. Clinical experience with this drug has revealed an extended spectrum of collateral effects and toxicities, from Grade 1 reaction, which does not require drug interruption, to severe reactions (Grade 3 and 4) called immune-related adverse events (irAE) that require prompt interruption of toxic therapy and often also the beginning of immunosuppressive drugs.

We present a case report about a female patient with severe irAEs caused by Pembrolizumab, prescribed for a metastatic skin melanoma. Although the side effects of this drug are largely known, we think that the acute multiple appearances of uncommon side effects on the same patient deserves a dissertation.

## 2. Case Presentation

We present the case of a 77-year-old female with a prior medical history of hypertension and type 2 diabetes treated with Enalapril/Lecarnidipine 20/10 mg once a day, Linagliptin/Metformin 2.5/850 mg after meals, as well as Pioglitazone, Acetylsalicylic acid, and Atorvastatin. In April 2021, she underwent the excision of a skin melanoma with axillary lymph node dissection and was subsequently started on Pembrolizumab therapy (second dose on August 2021). She had completed vaccination against SARS-CoV-2 in June 2021.

About 96 h after administration of Pembrolizumab, the outbreak of adverse effects began with diffuse myalgia, diarrhea, and asthenia, prompting her to seek medical assistance in our hospital. She arrived at the Emergency Department with obtundation and hypoglycemia, which was treated with infusion of Dextrose. Neurological exam revealed bilateral diplopia and blepharoptosis, severe ocular-paresis, and severe weakness of head and of proximal limb muscles (distal strength preserved). There was absence of dyspnea, dysphagia, and dysphonia. Initial laboratory tests showed severe metabolic acidosis with hyperkalemia and hyponatremia (pH 7.13, HCO_3_^-^ 8.5 mmol/L, Na^+^ 123 mmol/L, K^+^ 7.2 mmol/L, lactate 9.7 mmol/L), serum creatinine of 7.9 mg/dL, anuria, and elevated CK (peak value 3275 units/L) and myoglobin (persistently above 1000 units/L).

The patient was then admitted in the intensive care unit with acute renal failure and neurological adverse effects due to Pembrolizumab therapy. Treatment with high-dose steroids was initiated (Methylprednisolone 1 g qd for three days and then 40 mg tid). She additionally underwent renal replacement therapy (CVVHDF) and two sessions of plasmapheresis. During her ICU stay, there were no hemodynamical or respiratory problems of note. Furthermore, hepatic toxicity was excluded thanks to normal liver enzymes and normal upper abdominal echography.

After 48 h, an improvement in renal function was observed, with correction of metabolic acidosis and electrolyte abnormalities. There was a partial remission of blepharoptosis with persistent ophtalmoparesis and proximal limb weakness. Thyroid function tests revealed a state of hyperthyroidism with negative TSH receptor antibodies, leading us to think about endocrine toxicity, which is one of the described side effects.

As regards to radiology exams, a chest X-ray was performed during ICU stay, but it only showed a shaded parenchymal thickening of the right inferior pulmonary lobe in the absence of fever or cough. Moreover, two CT scans of the brain excluded brain stroke or tumors.

After a 4-day stay in the ICU, the patient was transferred from the ICU to the subacute care ward, where Methylprednisolone and Methimazole were gradually tapered. Neurological symptoms improved, and the patient started walking again. She was finally discharged from our hospital to a rehabilitation facility 20 days after admission.


**Recorded Parameters**

**At Admission**

**During the Stay**

**At Discharge**
Blood Pressure145/65 mmHg140/50 mmHg130/80 mmHgHeartbeat96 bpm80 bpm80 bpmBody Temperature36.4 °C36.0 °C36.0 °CGlycemia64 mg/dL122 mg/dL180 mg/dL

## 3. Discussion

Therapy with immune checkpoint inhibitors causes an unbalancing of the immune response that can cause autoimmune disorders [3,4,5], such as the severe adverse events described before. Treatment required the immediate interruption of the trigger drug, infusion of high-dose steroids, renal replacement therapy, plasmapheresis, and methimazole, as will be further discussed.

Our patient showed different types of toxicity, neurological, rheumatological, renal, and endocrine, with some manifestations more severe than the others but of equal importance.

Neurological toxicity appears typically within three months of therapy initiation and includes a wide range of pathologies [6,7,8,9,10,11] (headache, Guillain-Barré syndrome, myasthenia gravis, PRES, aseptic meningitis, transverse myelitis, pancerebellitis, autoimmune encephalitis, cranial, and peripheral neuropathies) [7,8,9,10]. ASCO (American Society of Clinical Oncology) guidelines and SITC (Society for Immunotherapy of Cancer) suggest treatment with high-dose glucocorticoids, intravenous immunoglobulins, and plasmapheresis [4,6].

Renal toxicity can cause an acute kidney injury (AKI) that is usually the result of a tubulointerstitial nephritis [12,13]. In our present case, it is important to consider that the patient was taking Metformin at home. The first ABG revealed a severe metabolic acidosis with a high anion gap, hyperkalemia, and high lactate: the AKI was therefore attributed to the overlapping of Pembrolizumab and Metformin toxicity. Immediate discontinuation of Metformin and ACE inhibitors was prescribed, and then patient underwent renal replacement therapy until an improvement in renal function was confirmed clinically (restoration of diuresis > 1 mL/kg/h) and with lab results. Continuous infusion of rapid-acting insulin was used to maintain an adequate level of plasma glucose. Plasmapheresis was performed to reduce the serum concentration of the drug, and a 12.000 mL of volume was processed.

Rheumatological toxicity can manifest frequently with a generalized myositis [14]. Our patient presented diffuse myositis upon admission. Sometimes the myositis can be associated with myocarditis, which can be fatal, but this was not the case for us, as ECG and echocardiogram were within normal limits.

Endocrine toxicity can be seen either in the form of hypothyroidism or hyperthyroidism [15], with the latter being less frequent. We were able to promptly identify this abnormality as the initial lab workup panel showed low TSH levels (0.034 uU/mL) and high FT4 levels (24 pg/mL) with negative TSH receptor antibodies. Methimazole therapy was started as soon as the results were made available and was maintained until discharge of the patient from our hospital.

## 4. Conclusions

Immune checkpoint inhibitors represent a new and important therapeutic weapon against cancer. Nevertheless, they can cause severe toxicities that put the patient at high risk and often require aggressive treatment such as high-dose glucocorticoids, plasmapheresis, and even admission in the ICU. The further clinical treatment of the metastatic tumor affecting the patient should undergo an oncological study in terms of risk–benefit and possible alternatives, because the occurrence of severe irAE with the necessity of high-dose steroids, in our opinion, clearly prevents restarting the therapy with the same drug.
